# Elongated Particles Show a Preferential Uptake in Invasive Cancer Cells

**DOI:** 10.3390/nano14231891

**Published:** 2024-11-25

**Authors:** Talya Cohen, Chalom Zemmour, Ora T. Cohen, Ofra Benny

**Affiliations:** Institute for Drug Research, The School of Pharmacy, Faculty of Medicine, The Hebrew University of Jerusalem, Jerusalem 9112001, Israel; talya.cohen2@mail.huji.ac.il (T.C.); chalom.zemmour@mail.huji.ac.il (C.Z.); ora.cohen3@mail.huji.ac.il (O.T.C.)

**Keywords:** cancer cells, PLGA particles, ellipsoid particles

## Abstract

Mechanically driven cellular preference for drug carriers can enhance selectivity in cancer therapy, underscoring the importance of understanding the physical aspects of particle uptake. In this study, it was hypothesized that elongated particles might be preferentially taken up by deformable, aggressive cancer cells compared to normal cells. Two film-stretching methods were tested for 0.8–2.4 μm polystyrene (PS) particles: one based on solubility in organic solvents and the other on heat-induced softening. The heat-induced method produced more homogenous particle batches, with a standard deviation in the particle aspect ratio of 0.42 compared to 0.91 in the solvent-based method. The ability of cells to engulf elongated PS particles versus spherical particles was assessed in two subsets of human melanoma A375 cells. In the more aggressive cancer cell subset (A375+), uptake of elongated PS particles increased by 10% compared to spherical particles. In contrast, the less aggressive subset (A375−) showed a 25% decrease in uptake of elongated particles. This resulted in an uptake ratio between A375+ and A375− that was 1.5 times higher for elongated PS particles than for spherical ones. To further demonstrate relevance to drug delivery, elongated paclitaxel-loaded biodegradable, slow-releasing poly(lactic-co-glycolic) acid (PLGA) particles were synthesized. No significant difference in cytotoxic effect was observed between A375+ and A375− cells treated with spherical drug-loaded particles. However, treatment with ellipsoidal particles led to a significantly enhanced cytotoxic effect in aggressive cells compared to less aggressive cells. These findings present promising directions for tailored cancer drug delivery and demonstrate the importance of particle physical properties in cellular uptake and drug delivery mechanisms.

## 1. Introduction

Cell deformability affects many biological processes, including cell division, mobility, and engulfment of particles. Cell deformation occurs in endocytosis and involves membrane bending [1,2,3], cytoskeleton stretching and turnover in cell spreading [4,5,6], and the formation of lamella structures in cell motility or phagocytic cups [7,8,9,10]. The process of phagocytosis is primarily carried out by specialized phagocytic cells, such as monocytes, macrophages, and neutrophils, which engulf and eliminate foreign entities like parasites, bacteria, dead cells, and cellular debris [11]. Along with the specific biological receptors that mediate phagocytosis [12,13], it has been shown that the mechanical state of macrophages, a key type of phagocyte, can also affect their capacity for particle uptake [14]. Beyond these professional phagocytes, many other cell types whose primary function is not phagocytosis (e.g., epithelial, endothelial, fibroblast, cancer, and mesenchymal cells) can also engage in particle uptake through a phagocytic-like process [15,16]. In these cells, particle internalization occurs independently of known specific receptors. Notably, cancer cells have been shown to efficiently engulf submicron- and micron-sized particles, with varying uptake depending on the cancer’s origin [16].

In endocytosis, which is an active cellular uptake process, cells invest energy to engulf nanoparticles, typically under 300 nm [17,18], in a process that involves membrane bending and encapsulation prior to internalization. At larger particle scales, above 300 nm, cell uptake occurs via a phagocytic-like mechanism that is independent of endocytosis mediators such as clathrin and caveolin [17,18]. In this process, the cell body distorts massively in a cytoskeleton-mediated manner. This process is initiated by the binding of a particle to the cell membrane and is followed by actin-driven particle engulfment [16,19].

A clear link between cell elasticity and malignancy was found for several cancer cell lines [20,21] and patient-derived cancer cells [19,22]. Our previous comprehensive study revealed a triangular correlation between cancer cell deformability, phagocytic potential, and malignancy [16]. This correlation suggests that cells with high inherent deformability (i.e., capacity to change their shape on the microscale), and reduced membrane tension can bend their membrane more easily to engulf nano or microparticles which are “difficult” to engulf by stiffer cells [23].

Cells have been shown to have an optimal range of particle shapes and sizes that they can effectively engulf, based on their specific mechanical properties [24,25,26,27]. Anisotropic particles and the level of asymmetry can affect the extent of cell uptake. While many studies compared the uptake of particles with various aspect ratios (ARs) in certain cells, the conclusions were inconsistent. Some studies have shown that elongated particles might have a higher chance of being internalized than spherical particles due to their increased contact area with the cell membrane [28,29]. In contrast, other studies have reported that particles with a higher AR are internalized to a lower extent than spherical particles [26,30]. However, these studies typically focus on comparing uptake in the same cell type. In our study, we address a critical gap by comparing how aggressive, deformable cells internalize elongated particles versus spherical particles, in contrast to non-aggressive, less deformable cells. Understanding these differences is particularly important because cellular mechanical properties, such as deformability, may significantly influence the internalization of particles with different shapes. This distinction has broad implications for the design of targeted therapies, as it can inform the development of particles optimized for specific cell types, such as those in tumors versus healthy tissues. For our cell models, human melanoma cell lines that were previously generated and characterized were used [16]. These cells were derived from the same original source and were sorted based on their phagocytic capacity. Cells referred to as A375 positive (A375+) were shown to be more aggressive and have higher phagocytic capacity [31], while A375 negative (A375−) cells were less aggressive and phagocytic, as previously detailed. Ex vivo, A375+ cells were more invasive in the spheroid collagen invasion assay and in vivo induced 64% larger tumors when injected into mice compared to A375− cells [16]. While these cell lines are from the same source and thus share many characteristics, the primary difference observed lies in their mechanical properties, phagocytic behavior, and overall aggressiveness, which contributed to the selection of these cells for our study.

We hypothesized that deformable, highly malignant cells would take up particles with a high AR more efficiently than stiffer, less malignant cells. To assess our hypothesis, we first established polystyrene (PS) stretching methods to produce particles with various ARs. Two particle stretching techniques were tested: one based on PS solubility in organic solvents and another involving heat. Then, the uptake difference between A375+ and A375− of elongated particles versus spherical particles was evaluated.

To further translate the differences in uptake into biological effects in drug delivery, we adapted the heat-induced stretching method from PS to biodegradable, poly(lactic-co-glycolic acid) (PLGA) particles loaded with paclitaxel. The viability of the aggressive deformable (A375+) cells treated with elongated and spherical paclitaxel-loaded PLGA particles was compared to the viability of their less aggressive counterparts (A375−).

## 2. Materials and Methods

### 2.1. Materials

Ester-terminated poly(lactic-*co*-glycolic) acid 50:50 (PLGA molecular weight (*M*_w_) 54,000–69,000, Sigma-Aldrich, St. Louis, MO, USA); Fluorescent Yellow Particles, 1% *w*/*v*, 0.7–0.9 µm (FP-0852-2) and Fluorescent Purple Particles, Medium Intensity, 1% *w*/*v*, 1.7–2.4 µm (FP-2062-2) polystyrene beads (Spherotech Inc., Lake Forest, IL, USA); polyvinyl alcohol (PVA *M*_w_~67,000, Sigma-Aldrich, USA); glycerol (Sigma-Aldrich, USA); tween 80 (Fisher BioReagent, Pittsburgh, PA, USA); 6-coumarin (Sigma-Aldrich, USA); dichloromethane (DCM) (Bio-Lab, Jerusalem, Israel); mineral oil (Sigma-Aldrich, USA); toluene (Bio-Lab, Israel); isopropyl alcohol (Daejung Chemicals, Siheung-si, Republic of Korea); minimum essential medium (MEM) eagle (Sartorius, Göttingen, Germany); Dulbecco’s phosphate-buffered saline (PBS) (Sigma-Aldrich, USA); paclitaxel (Signa-Aldrich, USA); and MTT (Sigma-Aldrich, USA).

### 2.2. Ellipsoidal Particle Preparation

#### 2.2.1. Preparation of Particle-Loaded Polyvinyl Alcohol (PVA) Films

PVA (5% *w*/*v*) was dissolved in 300 mL doubled-distilled water (DDW) at 85 °C. Glycerol 2% (*v*/*v*) was added to 8 mL of the 5% PVA solution to plasticize and reduce the glass transition temperature (*Tg*) of the films. A quantity of 80 µL of the stock solution of spherical PS particles were added to this mixture, and the films were dried on a 32 × 85 mm flat plastic surface (rectangle petri dish) at 37 °C for 24 h to a thickness of ~120 µm.

#### 2.2.2. Film Stretching

The films were typically cut into sections of 4 × 3 cm and their thicknesses were measured by a digital caliper. The films were mounted on an axial stretcher comprised of two aluminum blocks mounted on a screw, which when turned separated the blocks, and the films were stretched in hot oil, a hot oven, or toluene. Films with PS particles were stretched in toluene or an oven at 140 °C. In cases of toluene stretching, the film was immersed in toluene for 20 h before stretching and 30 min after stretching. After toluene immersion, the film was removed, air dried for 10 h, and soaked in isopropanol for 2 h to extract residual toluene. Strips of the film with embedded spherical PS particles were marked with square grid lines (1 cm × 1 cm) with an ink marker before stretching. After stretching, the grid rectangles were cut out from the film, and the particles were extracted and recovered separately according to their stretch ratio. In cases of heat-stretching, the film was placed on the stretcher in the oven that was preheated to 140 °C. After 5 min in the hot oven, the film was stretched while still in the oven and maintained in the oven for 5 additional minutes. Afterwards, the film was removed from the oven and allowed to cool for 20 min. Films with PLGA particles were stretched in hot mineral oil or an oven. The oil temperature was measured with an infrared camera and maintained between 55 and 65 °C. After stretching the films in the hot oil, the films were removed, allowed to cool in air, and washed with isopropanol to extract residual oil. When stretched in an oven, the oven temperature was kept at 75 °C, and the films were kept in the heated oven for 5 min before and after stretching.

#### 2.2.3. Particle Recovery from the Stretched Film

The PVA films were dissolved in an isopropanol–water mixture (3:7 *v*/*v*) under moderate heat. The particles were washed by centrifugation with DDW five times to remove all PVA residues from the surface of the particles.

### 2.3. Particle Elongation and AR Analysis

To verify particle morphology, dozens of particles were imaged using an inverted fluorescent microscope (Nikon Eclipse Ti2, Melville, NY, USA) or High-Resolution Scanning Electron Microscopy (SEM-HR, Extra-High-Resolution Scanning Electron Microscope, Zeiss Gemini 560, Jena, Germany). Particle dimensions and AR were measured using the NIS Element AR 5.11 Analysis software (Nikon confocal software) or ImageJ 1.53 software. Statistical data and error were analyzed on GraphPad Prism 9 (www.graphpad.com (accessed on 21 November 2024), San Diego, CA, USA).

### 2.4. Selectivity Tests

#### 2.4.1. Cell Culture

Experiments were performed using A375 primary human melanoma cells from ATCC (American Type Culture Collection, Manassas, VA, USA), and were mycoplasma-free. Before the experiments, the cells were seeded and incubated to 70–80% confluence on a 10 cm dish. The cells were maintained in MEM, and supplemented with 10% (*v*/*v*) fetal bovine serum, 1% pyruvate, 1% glutamine, 1% MEM vitamin solution, 1% antibiotic (streptomycin (10,000 µg/mL), and penicillin (10,000 units/mL) at 37 °C with 5% CO_2_.

#### 2.4.2. Cell Particle Uptake Assay

To measure the extent of particle internalization by cells, flow cytometry analysis was used (CytoFLEX flow cytometer, Brea, CA, USA). Unmodified spherical 0.8 μm polystyrene particles fluorescing at an excitation/emission of 590/620 nm were used in this assay, together with the particles that had undergone stretching. Cells were seeded in six-well plates (200,000 cells/well) and left for 24 h to adhere to the plates. Spherical and stretched PS particles were then added at the same concentration (0.8 µg/mL) to incubate with the cells overnight. Controls consisted of cells without particles incubated under the same conditions. After incubation, the cells were washed twice with phosphate-buffered saline (PBS) and detached using trypsin B. Cells were then centrifuged and fixed with 4% paraformaldehyde (PFA), washed again, and filtered through a 40 μm nylon mesh into FACS tubes to remove debris. Analyses were performed using the FlowJo V10 software.

### 2.5. PLGA Microparticle Fabrication Using a Microfluidic Device

PLGA microparticles were prepared using a microfluidic device as previously described (Micronit Microtechnologies, Enschede, The Netherland) [32]. The flow-focusing chip was made of durable borosilicate glass and the fluidic slide was made of polypropylene. Chip dimensions were 45 mm × 15 mm, and the channel width and depth were 100 and 20 µm, respectively. PLGA (1% *w*/*v*) and 6-coumarin (5 μg/mL) fluorophore probe were dissolved in DCM. This organic phase was flowed in the dispersed phase channel at a flow rate of 80 μL/min. The aqueous phase containing 1% PVA was flowed in the continuous phase channels at 150 or 250 μL/min flow rates. The solutions with the droplets were stirred for 3 h to ensure complete solvent evaporation. The particles were washed three times with DDW, by centrifuge at 5000 rpm for 5 min.

### 2.6. Spherical and Ellipsoidal Paclitaxel Loaded PLGA Particle Fabrication

Quantities of 100 mg of PLGA and 3 mg of paclitaxel were added to 2.5 mL DCM containing 6-coumarin (5 μg/mL). This solution was added dropwise to 10 mL of an aqueous solution containing 1% PVA while sonicating at 160 watts for 2 min using a probe sonicator (Sonic Rupture 400, OMNI international, Kennesaw, GA, USA), followed by 3 h of stirring for complete evaporation of the organic solvent. The particles were washed with DDW via centrifugation and cast into four films. Two films were stretched in the oven set to 75 °C, then allowed to cool in the air and dissolved in DDW. The particles were washed and resuspended with PBS. As a control, the two other films underwent the entire procedure, including exposure to 75 °C, except for the stretching step. As a control, spherical and ellipsoidal particles without drug were prepared by the same process.

### 2.7. Paclitaxel Loading Test

Paclitaxel entrapment efficiency and drug loading were estimated by dissolving the particles in acetonitrile. The concentration of paclitaxel was measured by High-Performance Liquid Chromatography (HPLC, SHIMADZU, Canby, OR, USA) using a C18 column, a mobile phase of 60:40 acetonitrile-DDW (0.1% TFA) at pH 7, and a detection wavelength of 214 nm. The retention time for the paclitaxel peak was 4.7 min. The entrapment efficiency was calculated by the following equation:

$$\%\;Entrapment\;Efficiency=\frac{Amount\;of\;entrappped\;drug}{Total\;drug\;added}\times 100$$

### 2.8. Cell Viability Assay

To assess the viability of cells treated with paclitaxel-loaded nanoparticles and investigate whether ellipsoidal particles demonstrate enhanced selectivity towards aggressive cancer cells compared to spherical particles, positive and negative A375 cells were seeded in a 96-well plate at a density of 5 × 10^3^ cells/well and incubated for 48 h. The treatments (drug-free and paclitaxel-loaded spherical and ellipsoidal PLGA particles) were introduced at identical particle concentrations and the plates were incubated for an additional 24 h. After incubation, MTT (Sigma-Aldrich, USA) was added (0.5 mg/mL) into each well for viability detection and incubated at 37 °C and 5% CO_2_ for 40 min. The absorbance was measured at 540 nm using a plate reader (Synergy HT Multi-Mode Microplate Reader, BioTek, Shoreline, WA, USA). In order to assess the effect of drug release from engulfed particles in the absence of drug-containing particles in the surrounding medium, we seeded an additional plate where the medium was replaced 24 h after the introduction of the drug-containing particles. Cell viability was assessed 24 h after the introduction of the particle-free medium, 48 h after initial particle incubation.

## 3. Results

### 3.1. Ellipsoid Polystyrene Particle Fabrication

**Figure 1:**
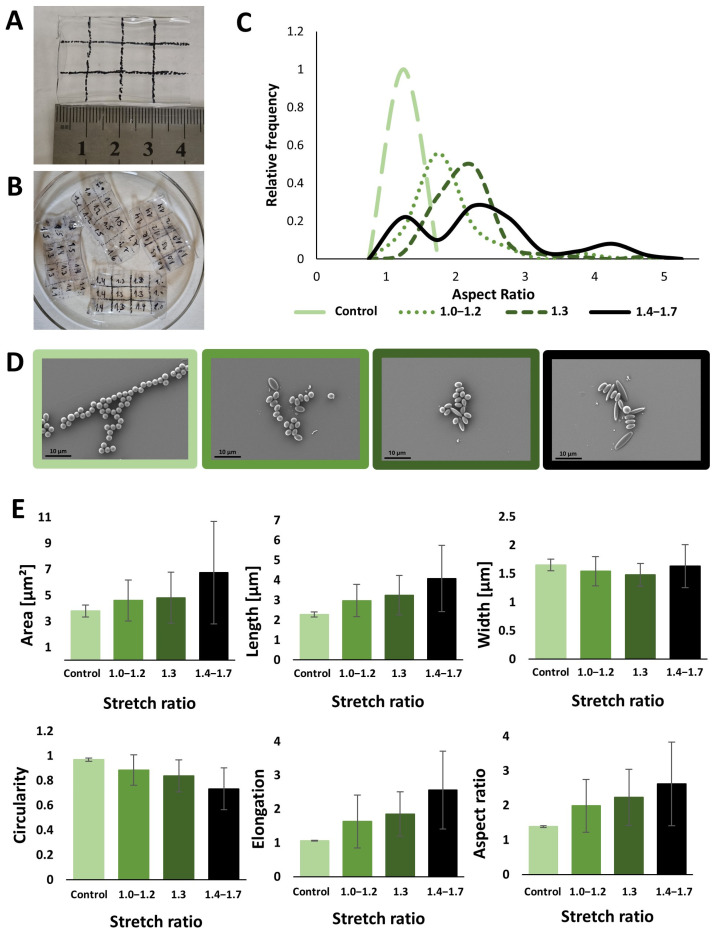
Solvent-induced polystyrene (PS) particle (2.4 µm) stretching. (**A**) Grid marking before stretching. The film was marked with a 1 cm × 1 cm grid. (**B**) Different stretch ratios (1.0–1.7) were measured on the film after stretching. (**C**) The aspect ratio distribution of the stretched PS particles in the different stretch ratios: 1.0–1.2, 1.3 and 1.4–1.7. (**D**) SEM images of the spherical and stretched PS particles. (**E**) Area, length, width, circularity, elongation, and aspect ratio mean of the spherical and stretched particles. Bars represent experimental means; error bars show standard deviation.

In our efforts to create ellipsoidal particles, the thin film-stretching method, a technique first reported by Ho, C.C. et al. was chosen [33]. When comparing two methods for PS particle stretching, namely liquefaction with toluene or through heating, it was found that toluene resulted in non-uniform stretching of the film. Therefore, films containing embedded spherical PS particles were marked with square grid lines (Figure 1A). After stretching, the grid rectangle lengths were measured and dissolved separately according to their stretch ratio: 1.0–1.2, 1.3, or 1.4–1.7 (Figure 1B). The graph depicting the AR distribution exhibits a rightward shift for the stretched particles, with a more pronounced shift observed for the stretch ratio of 1.3 compared to the stretch ratios of 1.0–1.2. Although the mean of the AR is highest for the stretch ratio 1.4–1.7, there is a large distribution in the AR of those particles (Figure 1C). Particle imaging by SEM revealed that as the stretch ratio increases, the area, length, elongation, and AR all increase, whereas the circularity decreases (Figure 1D,E).

**Figure 2:**
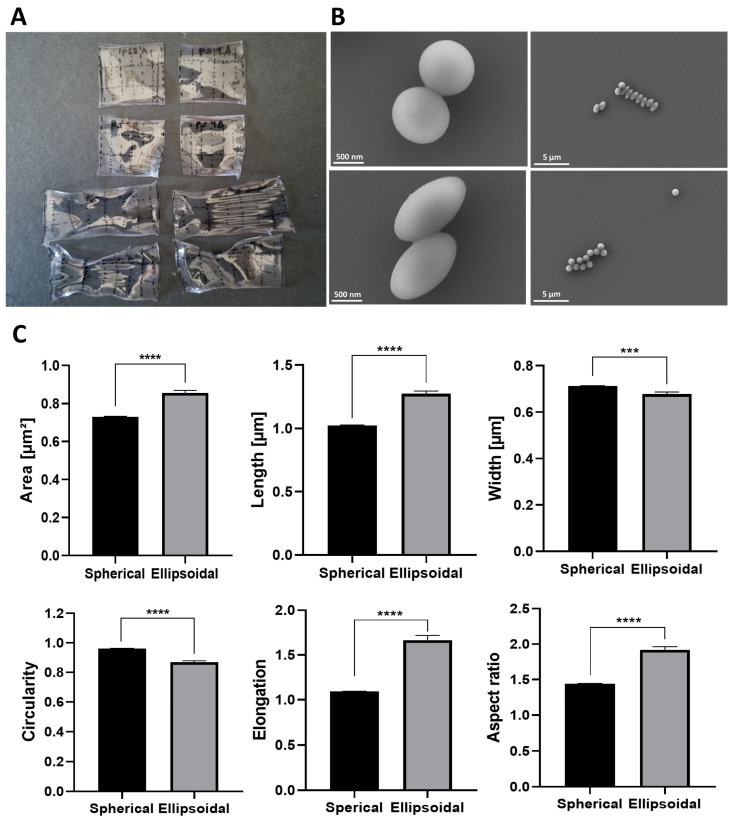
Thermo-induced polystyrene particle (0.8 µm) stretching. (**A**) The films of the non-stretched (top) and stretched (bottom) particles. The films were stretched to a stretch ratio of 2.5. (**B**) SEM images of the spherical (top) and ellipsoidal (bottom) PS particles. (**C**) Mean area, length, width, circularity, elongation, and aspect ratio of the spherical versus ellipsoidal particles. Bars represent experimental means; error bars show standard error (*n* = 76). Statistical significance was tested using an unpaired Student’s *t*-test, with the significance threshold set as *p* = 0.05 (***, *p* < 0.001; ****, *p* < 0.0001).

Thermal stretching was performed on films with embedded fluorescent 0.8 um PS particles, which were preheated to 140 °C and then stretched while maintained at this temperature. As a control, films with the same particle concentration underwent the entire procedure, including exposure to 140 °C, but without the stretching step (Figure 2A). The particles were imaged by SEM (Figure 2B) and the geometry of the stretched particles was analyzed and compared to the spherical particles. The stretched particles were ellipsoid in shape and carried an AR of ~2 (Figure 2C). Given that stretching under thermal conditions yielded superior results compared to solvent-based approaches, with consistent stretching across the entire film, this method resulted in higher particle homogeneity with a lower standard deviation of the particle AR (0.42 vs. 0.91) and obviated the necessity of grid marking. Since thermal stretching reduces the need for external agents and provides greater homogeneity in elongated particles, we decided to use this method for the remaining experiments in PS and PLGA particles.

### 3.2. Effect of AR on Particle Uptake

**Figure 3:**
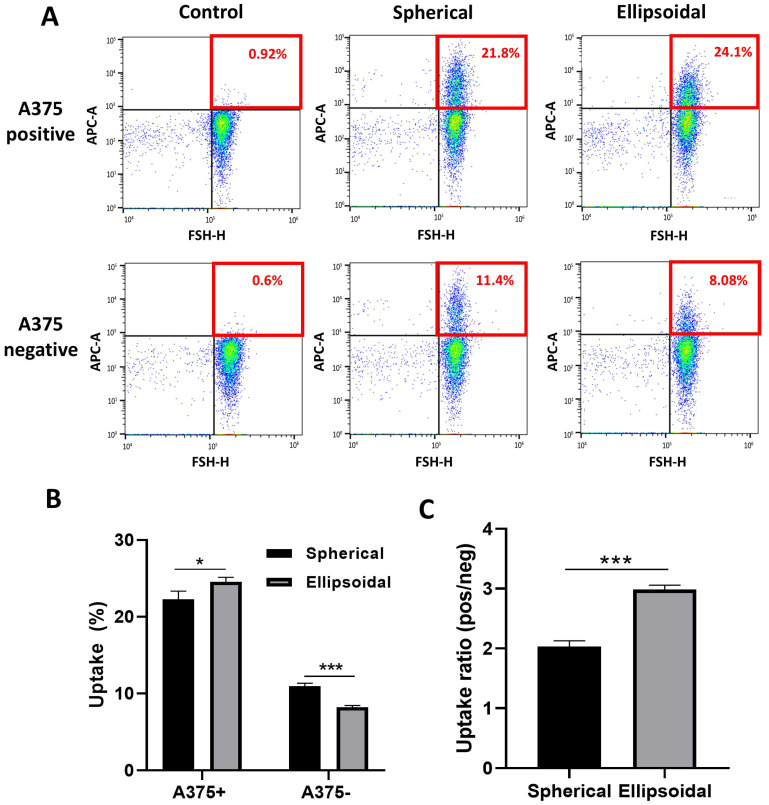
Flow cytometry analysis of cellular uptake of spherical and ellipsoidal polystyrene particles (0.8 µm) in A375 cells. (**A**) FACS plots and gating strategy used for uptake analysis. Control: Incubation with free-particle medium. (**B**) Uptake percentages measured by flow cytometry of spherical and ellipsoidal PS particles after incubation (overnight) in A375+ and A375− human melanoma cell lines. (**C**) The uptake ratio between A375+ to A375− for spherical and ellipsoidal particles. Control; no particles were added to the cells. Bars represent experimental means; error bars show standard error (*n* = 3). Statistical significance was tested using an unpaired Student’s *t*-test, with the significance threshold set as *p* = 0.05 (*, *p* < 0.05; ***, *p* < 0.001).

To determine whether particles with a higher AR preferentially target aggressive cancer cells, the 0.8 μm spherical (AR = 1.4) and stretched (AR = 2) particles were incubated overnight in A375+ and A375− cell lines. FACS was used to quantify uptake percentages, as depicted in Figure 3A. The findings demonstrate increased uptake of both spherical and ellipsoidal PS particles by A375+ cells compared to A375− cells. Interestingly, A375+ cells showed an increase of 10% (* *p* < 0.05) in the uptake of ellipsoidal particles compared to spherical ones, whereas in A375− the opposite trend was observed (a decrease of 25%, (*** *p* < 0.001)) (Figure 3B). This difference resulted in an uptake ratio between A375+ and A375− cells which was 1.5 times higher for ellipsoidal particles than for their spherical counterparts (Figure 3C).

### 3.3. Adapting the Stretching Protocol to Functional PLGA Particles

**Figure 4:**
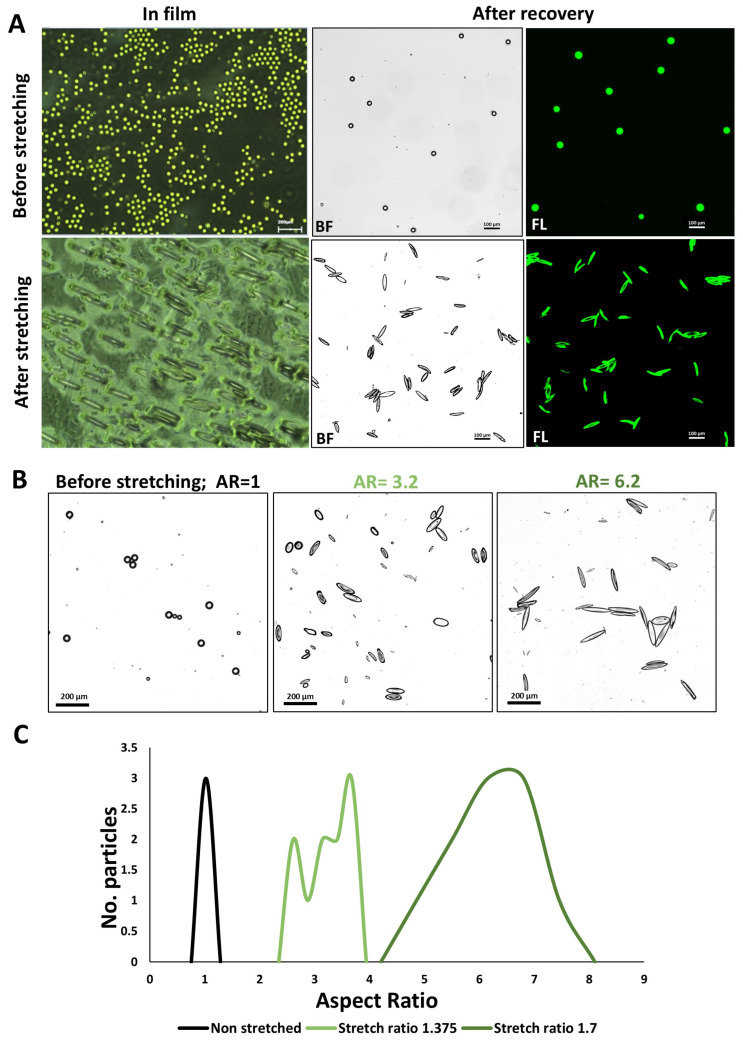
PLGA microparticle stretching. (**A**) 6-coumarin-loaded PLGA microparticles (~40 µm) (top) before and (bottom) after stretching, (left) inside the film, and (right) after recovery. (**B**) Bright field (BF) images of the PLGA particles before stretching and in two degrees of stretching (AR—average aspect ratio). (**C**) Aspect ratio distribution of the different stretch ratios.

In order to adapt our protocol to drug-loaded carriers, the heat-induced stretching protocol was applied to produce elongated PLGA particles. Figure 4A shows PLGA microparticles (~40 µm) loaded with 6-coumarin, fabricated through a single microfluidic device, and cast into a film. The images capture the particles before and after the stretching process within the film, along with their appearance post-recovery. Notably, the images highlight the uniformity of the newly acquired particle shape following stretching. In this case, 6-coumarin serves as a drug surrogate, illustrating the ability of the particles to retain a compound throughout the entire fabrication process, as seen by the fluorescence in the images.

When two films containing the same particles were stretched to different stretch ratios, 1.4 and 1.7, two populations of particles were obtained with average AR of 3.2 ± 0.4 and 6.2 ± 0.9, respectively (Figure 4B,C). The PLGA particles were produced using an artisanal method, while the PS particles were commercially purchased, resulting in greater variability in the final sizes of the PLGA particles in comparison with the PS particles. However, larger stretch ratios still resulted in larger ARs, underscoring our capability to regulate the degree of particle elongation.

### 3.4. Effect of Particle Shape on Cell Viability Based on Mechanical Targeting

**Figure 5:**
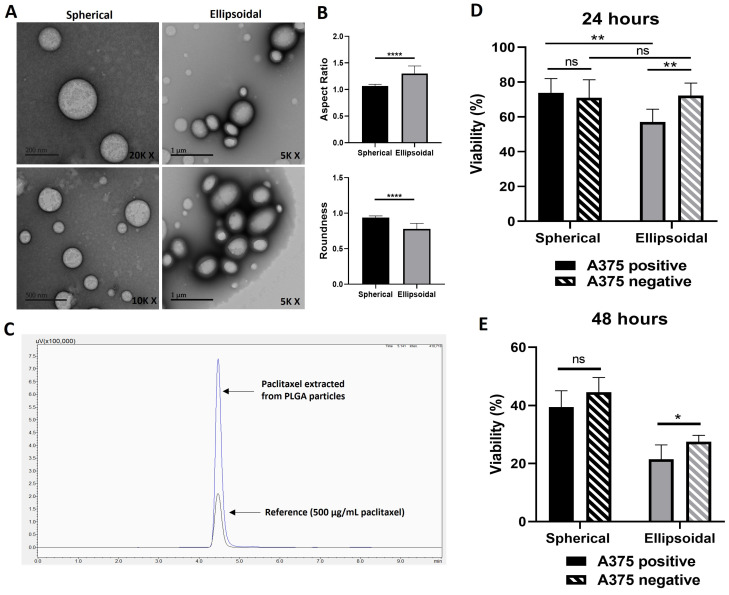
Selective killing of A375 positive cells via paclitaxel-loaded ellipsoidal particles. (**A**) TEM images of the spherical and ellipsoidal paclitaxel-loaded ~200 nm PLGA particles. (**B**) Mean aspect ratio and roundness of the spherical versus ellipsoidal particles. Analysis was performed using ImageJ. (**C**) Representative HPLC chromatograph which shows the results from HPLC purification experiment after paclitaxel extraction from PLGA particles. Reference shows peak obtained from paclitaxel standard solution (500 μg/mL), whereas the taller peak shows the amount of paclitaxel extracted from the PLGA particles. In this chromatograph, the peak corresponds to a concentration of 975 μg/mL of paclitaxel. (**D**) MTT assay results show A375 positive and negative cell viability after 24 h of incubation with spherical and ellipsoidal paclitaxel-loaded particles. (**E**) MTT results after 24 h incubation with paclitaxel-loaded particles followed by particle removal and an additional 24 h incubation in particle-free medium. Bars represent experimental means; error bars show standard error (*n* = 3). Statistical significance was tested using an unpaired Student’s *t*-test, with the significance threshold set as *p* = 0.05 (ns—nonsignificant, *p* > 0.05; *, *p* < 0.05; **, *p* < 0.01; ****, *p* < 0.0001).

To evaluate whether the selective uptake of ellipsoidal particles by A375+ cells leads to their targeted destruction when treated with chemotherapy-loaded particles, paclitaxel-loaded submicron particles (~200 nm) were synthesized in both spherical and ellipsoidal shapes. TEM images of these particles are displayed in Figure 5A. Analysis revealed that the spherical and ellipsoidal particles exhibited ARs of 1.0 ± 0.02 and 1.3 ± 0.14, and roundness values of 0.93 ± 0.02 and 0.78 ± 0.7, respectively (Figure 5B). The entrapment efficiency of paclitaxel within the particles was 32.5% as determined by HPLC (Figure 5C).

Following a 24 h incubation of both A375+ and A375− cells with the paclitaxel-loaded particles, MTT assays were conducted to evaluate cell viability immediately following the incubation period (Figure 5D), as well as after an additional 24 h of incubation in particle-free medium, after washing (Figure 5E). Figure 5D shows the short-term effect of the drug-loaded particles, while Figure 5E demonstrates the impact of drug release after particle internalization by the cells. In both scenarios, no significant differences (*p* = 0.05) in the viability of A375 positive and negative cells were observed when spherical particles were used. However, when treated with ellipsoidal particles, a significantly enhanced cytotoxic effect (** *p* < 0.01) was seen in the aggressive cells (viability of 57%) compared to the less aggressive cells (viability of 72%) after 24 h of incubation. This suggests that changing the particle shape from spheroidal to ellipsoidal enhanced the selectivity of the drug towards aggressive cancer cells, potentially due to mechanically driven cellular preference.

## 4. Discussion

Mechanical targeting is emerging as an important avenue in drug delivery, particularly in cancer therapy. Understanding the physical aspects of particle uptake by cancer cells may contribute to enhancing cell targeting and selectivity of drug delivery systems and could potentially be a consideration for precision nano-therapy. These mechanical processes involve intricate interactions between the physical properties of particles, including size, shape, and stiffness, and the biomechanical properties of the cell that are affected by membrane fluidity, viscoelasticity, and cytoskeleton dynamics. At submicron scales, the scales relevant to our study, uptake mechanisms are mainly “phagocytic-like” mechanisms, which are mainly affected by cell deformability. In this study, we focused on the effects of elongated versus spherical particles on the uptake of aggressive deformable cells.

The technique for controlling particle shape and AR has been demonstrated across various applications and research areas, including particle biodistribution [34], macrophage uptake [35], blood circulation and flow forces [36], extravasation rate [37], and binding activity [38]. However, unlike most studies in this field, our study focuses on the effects at the single-cell level rather than the tissue level, which involves many other physiological considerations.

Several approaches for creating non-spherical particles, including both top-down and bottom-up methods, have been reported in the literature [33,39,40,41,42]. However, many methods are not sufficiently robust and may not be suitable for drug-loaded carriers. We chose to employ the film-stretching approach [33] as we found it had the best capability to optimize the production of anisotropic particles across different sizes and stretch levels using a variety of polymers.

When comparing two methods for stretching film, it was found that heating the film above the *Tg* of the polymeric particles using a preheated oven yielded superior results compared to using a solvent, such as toluene, as a liquefying agent. The films stretched under heat displayed superior homogeneity and uniformity, leading to enhanced particle quality. Notably, this method eliminates the necessity for external agents like solvents, signifying a more straightforward and resource-efficient process.

Moreover, using an oven for heat convection, as opposed to the predominantly utilized hot mineral oil as reported in previous studies [33,43], was favored due to safety concerns associated with dealing with oils at high temperatures. For example, Hol. C et al. showed that when the oil was heated to 200 °C, the desired effect was achieved [33]. Furthermore, in the oven heating, no residual oil was left on the films at the end of the process. Hence, for the cell uptake experiment, we used the oil-free method. In the context of drug delivery, it should be noted that the thermal method is applicable only in cases where the active compound is not sensitive to high temperatures. While some drugs may lose activity under high temperatures, such as diphenhydramine, midazolam, and fentanyl, certain drugs remain stable in extreme temperatures and are compatible with processes requiring heat, such as morphine and ondansetron [44]. In our case, we used paclitaxel as a model chemotherapy, which exhibits good stability even when subjected to elevated temperatures [45].

Previous studies that have investigated the impact of anisotropic particle AR on uptake within specific cell types yielded inconsistent results regarding whether higher AR enhances or diminishes uptake. Most research carried out on this topic was performed in professional phagocytic immune cells. For example, the effects of ellipsoidal particles in macrophages in the range of 6–10 μm poly(ε-caprolactone) (PCL) colloids were shown to be shape- and time-dependent. The difference between the phagocytosis of the spheroids and the ellipsoids was more evident in the 6 μm particles and at early incubation times [46].

Indeed, in many examples, it was shown that non-spherical ellipsoidal polymer particles showed reduced engulfment compared to spherical ones. For example, Florez. et al. found that stretched polymeric nanoparticles with ARs of 2.3, 4, and 5.9 showed reduced uptake compared to spherical nanoparticles by human mesenchymal stromal and HeLa cells [30]. In contrast, Parakhonskiy et al. showed that the internalization rate of calcium carbonate particles by HeLa cells is increased with an increased AR [28]. Rather than focusing on the extent of cell internalization with particle elongation level, we specifically explored whether particle elongation might provide specificity towards highly aggressive and deformable cancer cells.

The engulfment and uptake of particles by cells require a sufficiently large area of cell–particle adhesion to reduce free energy while minimizing cell morphological changes, which could increase free energy. It was shown that cells with higher malignancy and metastatic potential exhibit greater elasticity and adhesion compared to normal or non-aggressive cells. For example, primary prostate cancer cells (PC3M-P) were found to be less elastic and adhesive than a metastatic subpopulation of these cells (PC3M-LN4) [16]. From a physical point of view, elastic cell deformation incurs a smaller energetic penalty, and the gain in adhesion energy is more significant, leading to increased uptake capacity of ellipsoidal particles. In contrast, the less aggressive, stiffer cells invest considerable cell energy in taking up elongated particles due to the need for extensive cytoskeleton remodeling for cell deformation and particle wrapping, in contrast to the uptake of spherical particles. This is reflected in the uptake behavior we observed in aggressive A375+ cells versus their less aggressive A375− counterparts.

The effect of the shape on cell uptake also yields potential ramifications on the biological effects of loaded drugs. Our findings confirmed that modifying the shape of drug-loaded particles to ellipsoids, without affecting drug content per particle, enhances the efficacy of chemotherapy in eliminating A375+ cells while having a smaller impact on A375− cells. This selective destruction of aggressive deformable cancer cells highlights the potential for mechanical-based targeting strategies in cancer therapy. Since the uptake was evaluated by flow cytometry as the percentage of cells that underwent uptake, the biological effect is likely due to an increase in the number of cells that took up one or more particles in a specific cell population.

The greater capability of aggressive and deformable cancer cells to preferentially uptake particles with a high AR, and therefore selectively be eradicated, highlights the potential of ellipsoidal particles to serve as candidates for targeted drug delivery in various biomedical applications. In addition to increasing the uptake of the drug carrier by target cells, the increased energetic cost of uptake by non-target cells can minimize unwanted side effects. This is particularly important in cancer therapies where the ability to selectively target and deliver drugs to malignant, more elastic cells could significantly improve therapeutic outcomes. However, it should be noted that our experiments were conducted at the single-cell level and should be further explored in a physiological context, where other considerations such as biodistribution, pharmacokinetics, and tissue barriers need to be considered.

Beyond cancer, this principle may also be applied to other areas of biomedical engineering, including tissue engineering and regenerative medicine, where cell deformation and particle uptake play critical roles in scaffolding, cell regeneration, and controlled delivery of bioactive molecules. The ability to selectively target specific cell types by exploiting their unique mechanical properties can also be valuable for the development of more precise immunotherapies, where particle shape may influence the interaction between immune cells and nanoparticles designed for vaccination or cancer immunotherapy. In addition to enhancing drug delivery, the increased energetic cost for non-target cells may reduce off-target effects, thus minimizing side effects associated with conventional treatments.

## 5. Conclusions

This study demonstrates the importance of particle shape in cell uptake and the variabilities between cells. We show that drug-loaded particles can be tuned to selectively interact with more aggressive cancer cell subtypes based on physical parameters. These results hold considerable promise for improving cancer treatment outcomes. The ability to selectively target and destroy aggressive cancer cells while sparing healthy cells is a significant advancement in the field of oncology. Tuning the physical properties of nano- and microparticles and considering the biomechanical properties of cancer cells may offer a new avenue for improving the efficacy and specificity of chemotherapy.

## Data Availability

The data presented in this study are available on request from the corresponding author.

## References

[1] Zhdanov V.P. (2013). Physical aspects of the initial phase of endocytosis. Phys. Rev. E.

[2] Gao H., Shi W., Freund L.B. (2005). Mechanics of Receptor-Mediated Endocytosis. Proc. Natl. Acad. Sci. USA.

[3] McMahon H.T., Gallop J.L. (2005). Membrane curvature and mechanisms of dynamic cell membrane remodelling. Nature.

[4] Brill-Karniely Y., Nisenholz N., Rajendran K., Dang Q., Krishnan R., Zemel A. (2014). Dynamics of cell area and force during spreading. Biophys. J..

[5] Raucher D., Sheetz M.P. (2000). Cell Spreading and Lamellipodial Extension Rate Is Regulated by Membrane Tension. J. Cell Biol..

[6] Dubin-Thaler B.J., Hofman J.M., Cai Y., Xenias H., Spielman I., Shneidman A.V., David L.A., Döbereiner H.G., Wiggins C.H., Sheetz M.P. (2008). Quantification of cell edge velocities and traction forces reveals distinct motility modules during cell spreading. PLoS ONE.

[7] Pacheco P., White D., Sulchek T. (2013). Effects of Microparticle Size and Fc Density on Macrophage Phagocytosis. PLoS ONE.

[8] Herant M., Heinrich V., Dembo M. (2006). Mechanics of neurophil phagocytosis: Experiments and quantitative models. J. Cell Sci..

[9] Ponti A., Machacek M., Gupton S.L., Waterman-Storer C.M., Danuser G. (2004). Two distinct actin networks drive the protrusion of migrating cells. Science.

[10] Cramer L.P. (2010). Forming the cell rear first: Breaking cell symmetry to trigger directed cell migration. Nat. Cell Biol..

[11] Feldman M.B., Vyas J.M., Mansour M.K. (2019). It takes a village: Phagocytes play a central role in fungal immunity. Semin. Cell Dev. Biol..

[12] Miyanishi M., Tada K., Koike M., Uchiyama Y., Kitamura T., Nagata S. (2007). Identification of Tim4 as a phosphatidylserine receptor. Nature.

[13] Li W. (2012). Eat-me signals: Keys to molecular phagocyte biology and ‘Appetite’ control. J. Cell. Physiol..

[14] Patel N.R., Bole M., Chen C., Hardin C.C., Kho A.T., Mih J., Deng L., Butler J., Tschumperlin D., Fredberg J.J. (2012). Cell Elasticity Determines Macrophage Function. PLoS ONE.

[15] Couzinet S., Cejas E., Schittny J., Deplazes P., Weber R., Zimmerli S. (2000). Phagocytic Uptake of Encephalitozoon Cuniculi by Nonprofessional Phagocytes. Infect. Immun..

[16] Brill-Karniely Y., Dror D., Duanis-Assaf T., Goldstein Y., Schwob O., Millo T., Orehov N., Stern T., Jaber M., Loyfer N. (2020). Triangular correlation (TrC) between cancer aggressiveness, cell uptake capability, and cell deformability. Sci. Adv..

[17] Tischenko K., Brill-Karniely Y., Steinberg E., Segev-Yekutiel H., Benny O. (2023). Surface physical cues mediate the uptake of foreign particles by cancer cells. APL Bioeng..

[18] Manzanares D., Ceña V. (2020). Endocytosis: The nanoparticle and submicron nanocompounds gateway into the cell. Pharmaceutics.

[19] Quan F.S., Kim K.S. (2016). Medical applications of the intrinsic mechanical properties of single cells. Acta Biochim. Biophys. Sin..

[20] Suresh S. (2007). Biomechanics and biophysics of cancer cells. Acta Biomater..

[21] Seyfried T.N., Huysentruyt L.C. (2013). On the Origin of Cancer Metastasis. Crit. Rev. Oncog..

[22] Cross S.E., Jin Y.S., Rao J., Gimzewski J.K. (2007). Nanomechanical analysis of cells from cancer patients. Nat. Nanotechnol..

[23] Joseph J.G., Liu A.P. (2020). Mechanical Regulation of Endocytosis: New Insights and Recent Advances. Adv. Biosyst..

[24] Zheng M., Yu J. (2016). The effect of particle shape and size on cellular uptake. Drug Deliv. Transl. Res..

[25] He C., Hu Y., Yin L., Tang C., Yin C. (2010). Effects of particle size and surface charge on cellular uptake and biodistribution of polymeric nanoparticles. Biomaterials.

[26] Chithrani B.D., Ghazani A.A., Chan W.C.W. (2006). Determining the size and shape dependence of gold nanoparticle uptake into mammalian cells. Nano Lett..

[27] Dasgupta S., Auth T., Gompper G. (2014). Shape and orientation matter for the cellular uptake of nonspherical particles. Nano Lett..

[28] Parakhonskiy B., Zyuzin M.V., Yashchenok A., Carregal-Romero S., Rejman J., Möhwald H., Parak W.J., Skirtach A.G. (2015). The influence of the size and aspect ratio of anisotropic, porous CaCO_3_ particles on their uptake by cells. J. Nanobiotechnol..

[29] Brill-Karniely Y., Schwob O., Benny O. (2022). The aspect ratio effect on the cytotoxicity of inert nano-particles flips depending on particle thickness, and is one of the reasons for the literature inconsistency. Nanoscale Adv..

[30] Florez L., Herrmann C., Cramer J.M., Hauser C.P., Koynov K., Landfester K., Crespy D., Mailänder V. (2012). How shape influences uptake: Interactions of anisotropic polymer nanoparticles and human mesenchymal stem cells. Small.

[31] Goldstein Y., Cohen O.T., Wald O., Bavli D., Kaplan T., Benny O. (2024). Particle uptake in cancer cells can predict malignancy and drug resistance using machine learning. Sci. Adv..

[32] Amoyav B., Benny O. (2018). Controlled and tunable polymer particles’ production using a single microfluidic device. Appl. Nanosci..

[33] Ho C.C., Keller A., Odell J.A., Ottewill R.H. (1993). Preparation of Monodisperse Ellipsoidal Polystyrene Particles. Colloid. Polym. Sci..

[34] Arnida M.M., Ray A., Peterson C.M., Ghandehari H. (2011). Geometry and surface characteristics of gold nanoparticles influence their biodistribution and uptake by macrophages. Eur. J. Pharm. Biopharm..

[35] Champion J.A., Mitragotri S. (2006). Role of Target Geometry in Phagocytosis. Proc. Natl. Acad. Sci. USA.

[36] Gavze E., Shapiro M. (1997). Particles in a shear flow near a solid wall: Effect of nonsphericity on forces and velocities. Int. J. Multiph. Flow..

[37] Smith B.R., Kempen P., Bouley D., Xu A., Liu Z., Melosh N., Dai H., Sinclair R., Gambhir S.S. (2012). Shape matters: Intravital microscopy reveals surprising geometrical dependence for nanoparticles in tumor models of extravasation. Nano Lett..

[38] Decuzzi P., Ferrari M. (2008). The receptor-mediated endocytosis of nonspherical particles. Biophys. J..

[39] Rolland J.P., Maynor B.W., Euliss L.E., Exner A.E., Denison G.M., DeSimone J.M. (2005). Direct fabrication and harvesting of monodisperse, shape-specific nanobiomaterials. J. Am. Chem. Soc..

[40] Buyukserin F., Aryal M., Gao J., Hu W. (2009). Fabrication of polymeric nanorods using bilayer nanoimprint lithography. Small.

[41] Jang S.G., Audus D.J., Klinger D., Krogstad D.V., Kim B.J., Cameron A., Kim S.-W., Delaney K.T., Hur S.-M., Killops K.L. (2013). Striped, ellipsoidal particles by controlled assembly of diblock copolymers. J. Am. Chem. Soc..

[42] Mondiot F., Wang X., De Pablo J.J., Abbott N.L. (2013). Liquid crystal-based emulsions for synthesis of spherical and non-spherical particles with chemical patches. J. Am. Chem. Soc..

[43] Champion J.A., Katare Y.K., Mitragotri S. (2007). Making polymeric micro- and nanoparticles of complex shapes. Proc. Natl. Acad. Sci. USA.

[44] Armenian P., Campagne D., Stroh G., Tallman C.I., Zeng W.Z.D., Lin T., Gerona R.R. (2017). Hot and Cold Drugs: National Park Service Medication Stability at the Extremes of Temperature. Prehospital Emerg. Care.

[45] Shanmugam S., Park J.-H., Chi S.-C., Yong C.S., Choi H.-G., Woo J.S. (2011). Physicochemical stability, pharmacokinetic, and biodistribution evaluation of paclitaxel solid dispersion prepared using supercritical antisolvent process. Drug Dev. Ind. Pharm..

[46] Friess F., Roch T., Seifert B., Lendlein A., Wischke C. (2019). Phagocytosis of spherical and ellipsoidal micronetwork colloids from crosslinked poly(ε-caprolactone). Int. J. Pharm..

